# Efficacy and safety of sacituzumab govitecan in metastatic breast cancer: a single-center retrospective cohort study

**DOI:** 10.3389/fonc.2026.1951068

**Published:** 2026-10-13

**Authors:** Orcun Can, Osman Gökhan Demir

**Affiliations:** Department of Medical Oncology, Acibadem Mehmet Ali Aydinlar University, Acibadem Maslak Hospital, Istanbul, Türkiye

**Keywords:** CNS metastases, metastatic breast cancer, progression-free survival, sacituzumab govitecan, triple-negative breast cancer

## Abstract

Metastatic breast cancer (MBC) still remains incurable after advances in the field of systemic therapy. Sacituzumab govitecan (SG) is an antibody-drug conjugate that works against TROP-2 and clinical studies have shown its effectiveness in patients who have received multiple lines of treatment prior to its administration. However, the number of real-world studies is still limited in this field. The present study was performed retrospectively in one center and aimed at evaluating 48 women with MBC treated with SG in the period of December 2022 until March 2024. Clinical characteristics, toxicity of the treatment (CTCAE v5.0), pathology of progression-free survival (PFS) and overall survival (OS) were studied. The median age of the patients was 50.5 years, with 68.8% of them having triple-negative breast cancer and 31.2% having positive hormone receptors and negative HER2. The percentage of the women having metastases in their central nervous system is 31.2%. Three was the median number of treatment lines undertaken prior to the trial. According to the data obtained in the course of the study, median PFS and OS are equal to 3.9 and 9.85 months respectively. At the same time, the percentage of the patients experiencing Grade 3–4 side effects is equal to 39.6%, with neutropenia being the most characteristic for SG use. Dose adjustments were made for 41.7% of the patients and 12.5% had to stop treatment. Overall, SG demonstrated clinical activity in this real-world cohort, although Grade 3–4 adverse events and treatment modifications were relatively frequent, highlighting the importance of close monitoring and supportive care.

## 1 Introduction

Metastatic breast cancer (MBC) continues to represent a significant global health challenge, accounting for the majority of breast cancer-related mortality despite substantial advancements in early detection and localized treatment modalities (1) (2),. Although therapeutic innovations have dramatically improved outcomes for early-stage breast cancer, MBC remains incurable, with a median overall survival ranging between 12 and 36 months depending on molecular subtype, treatment history, and patient characteristics (3) (4),.

Triple-negative breast cancer (TNBC) and hormone receptor-positive/HER2-negative (HR+/HER2−) subtypes comprise the largest subsets of MBC and present distinct therapeutic challenges (5). TNBC, in particular, is associated with aggressive clinical behavior, limited therapeutic targets, and high rates of visceral and central nervous system (CNS) metastases. The lack of targetable hormone receptors or HER2 amplification restricts treatment options primarily to cytotoxic chemotherapy and, more recently, immunotherapy combinations. Similarly, resistance to endocrine therapy and chemotherapy regimens limits durable disease control in HR+/HER2− patients, particularly after multiple lines of treatment, necessitating novel therapeutic strategies (6) (7, 8).

Antibody-drug conjugates (ADCs) have emerged as a promising class of targeted therapies that combine the specificity of tumor-targeting antibodies with the potency of cytotoxic agents. This approach enables selective delivery of highly toxic payloads directly to cancer cells while potentially minimizing systemic exposure and associated toxicities (9). *Sacituzumab govitecan* (SG, Trodelvy^®^) represents a novel ADC that targets TROP-2 (trophoblast cell-surface antigen 2), a transmembrane glycoprotein highly expressed in multiple epithelial malignancies, including breast cancer (10) (11),.

The mechanism of action of SG involves binding to TROP-2-expressing cells, followed by internalization and release of SN-38, the active metabolite of irinotecan, directly into the tumor microenvironment (11). This targeted delivery system enables selective cytotoxicity with improved therapeutic indices compared to conventional chemotherapy. Additionally, the released SN-38 can exert a bystander effect on neighboring cancer cells, potentially overcoming tumor heterogeneity in TROP-2 expression (11, 12).

Pivotal clinical trials, including the phase 3 ASCENT study in TNBC and TROPiCS-02 in HR+/HER2− breast cancer, have demonstrated significant clinical benefits of SG in heavily pretreated patient populations, leading to regulatory approvals for both indications (7, 13). The ASCENT trial showed a median progression-free survival (PFS) of 5.6 months and overall survival (OS) of 12.1 months in patients with metastatic TNBC who had received at least two prior therapies.

However, real-world data validating the safety and efficacy of SG across diverse patient populations, particularly those with brain metastases, varying performance status, or different TROP-2 expression levels, remain limited (14, 15). Furthermore, the translation of clinical trial efficacy to routine oncology practice requires validation in less selected patient cohorts that better represent the complexity of real-world MBC populations.

Our retrospective cohort study aimed to assess the real-world clinical outcomes, safety profile, and tolerability of SG in heavily pretreated MBC patients at a single academic medical center (15). We further explored treatment responses across molecular subtypes, evaluated the impact of CNS involvement on safety and efficacy outcomes, and compared our findings with published clinical trial data to provide insights into the broader applicability of SG in routine clinical practice (16, 17).

## 2 Results

### 2.1 Patient characteristics and treatment history

A total of 48 patients with metastatic breast cancer who received sacituzumab govitecan were included in this analysis. The baseline demographic and clinical characteristics are summarized in Table A.1. The median age was 50.5 years (range: 31–69 years), with the majority of patients (68.8%) being postmenopausal. Two-thirds of patients (64.6%) presented with *de novo* metastatic disease, while the remainder developed metastatic recurrence following treatment for early-stage breast cancer. **Figure 1** showing age distribution.

**Figure 1:**
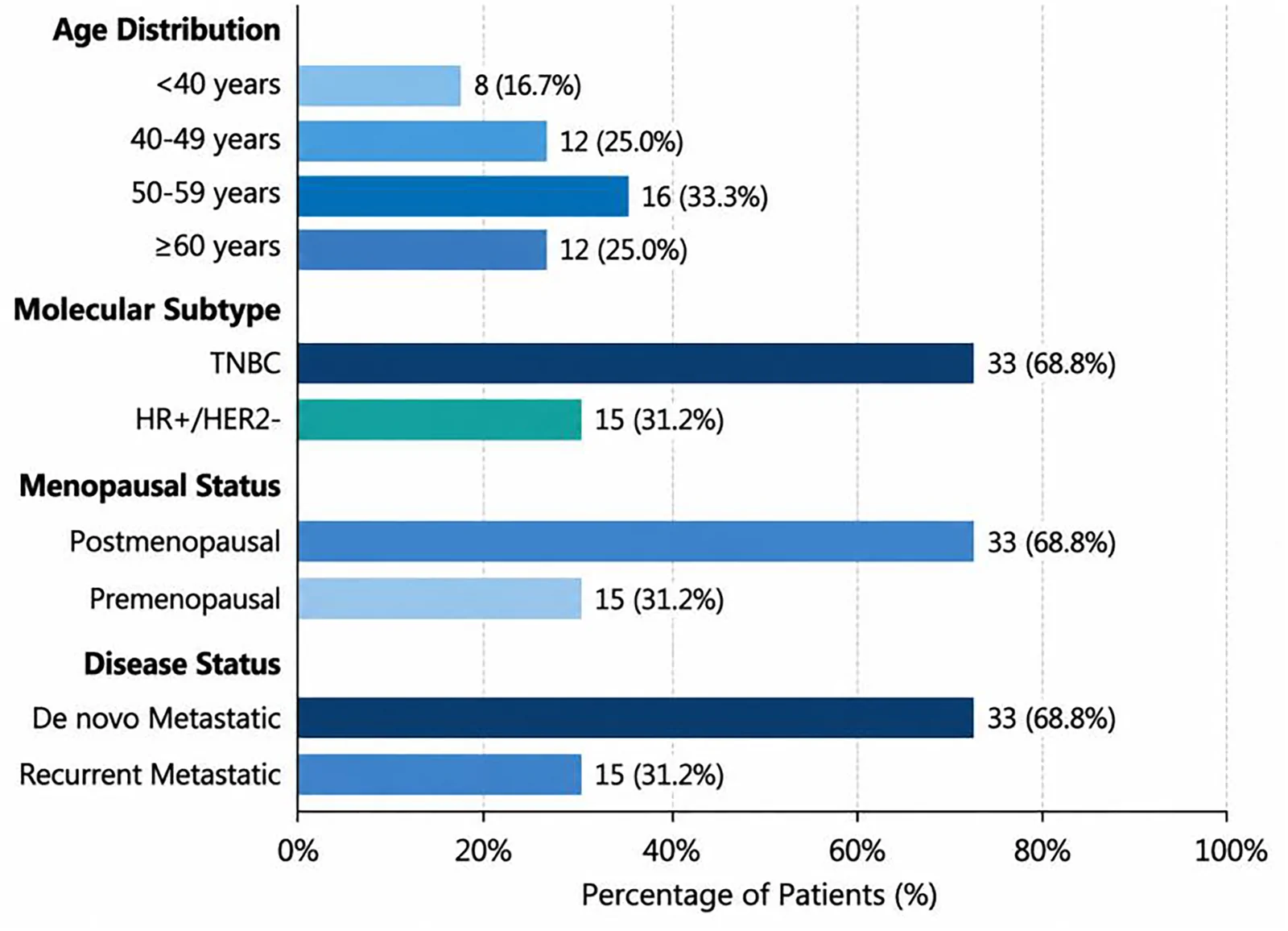
Patient demographics bar chart.

Molecular subtype distribution revealed that 33 patients (68.8%) had triple-negative breast cancer, while 15 patients (31.2%) had hormone receptor-positive/HER2-negative disease. Central nervous system metastases were present in 15 patients (31.2%) at the time of SG initiation, representing a substantial proportion of patients with this high-risk feature typically underrepresented in clinical trials.

The patient population was heavily pretreated, reflecting real-world clinical practice patterns. The median number of prior treatment lines in the metastatic setting was 3 (range: 1-6). Prior treatment exposures included PARP inhibitors in 13 patients (27.1%) and immunotherapy combinations in 24 patients (50.0%). All patients with TNBC had received prior anthracycline and taxane-based chemotherapy, while patients with HR+/HER2− disease had received multiple lines of endocrine therapy and chemotherapy. **Figure 2** displaying median number of prior treatment lines and percentage of patients.

**Figure 2:**
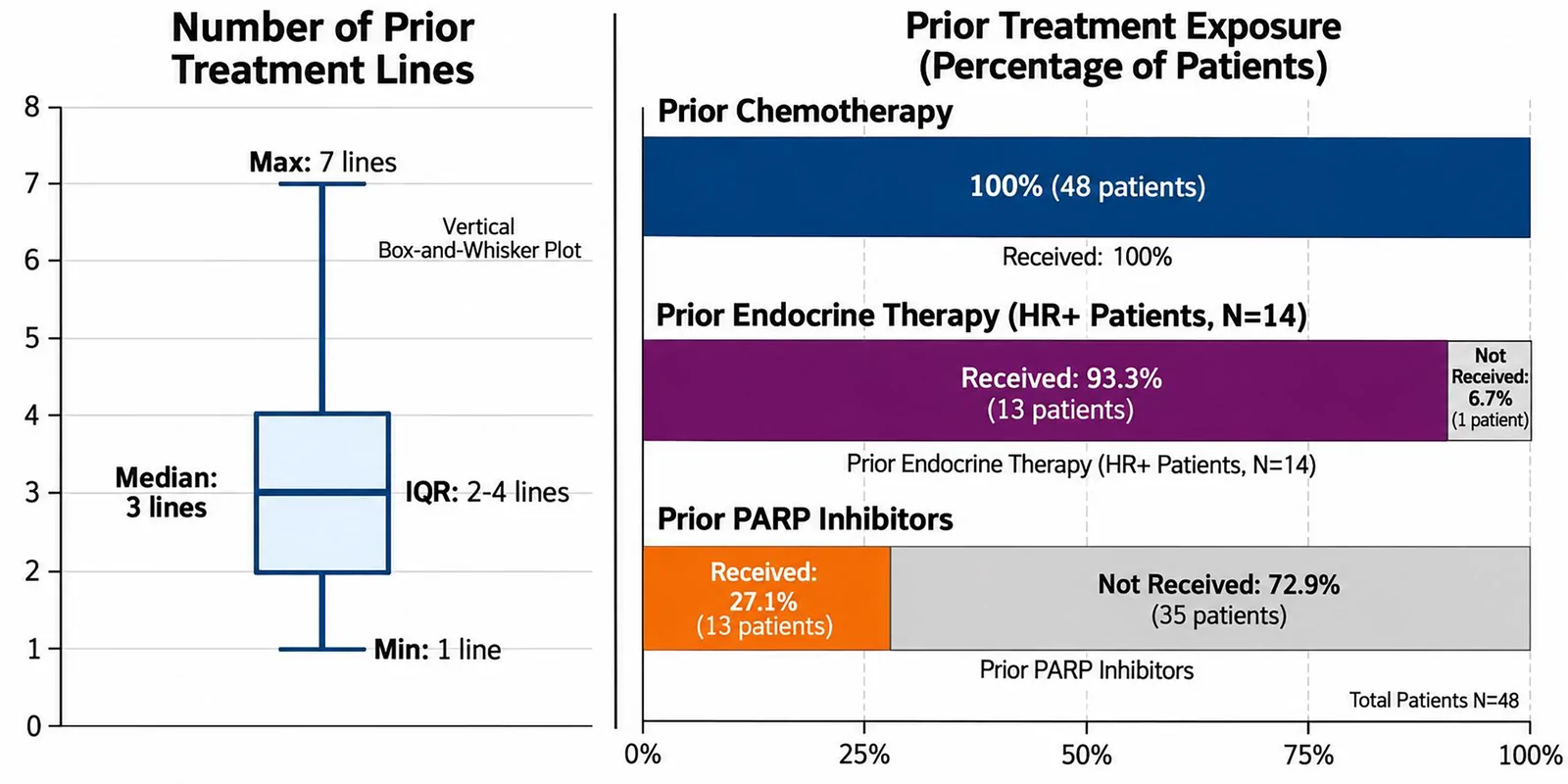
Prior treatment exposure chart.

### 2.2 Efficacy outcomes

At a median follow-up of 12.5 months (range: 3.2-18.7), survival outcomes demonstrated clinically meaningful activity of sacituzumab govitecan in this heavily pretreated population. The median progression-free survival for the entire cohort was 3.9 months (95% CI: 2.1-5.7), with a range of 0-20.1 months. Six patients (12.5%) achieved progression-free survival exceeding 12 months, indicating durable disease control in a subset of patients. **Figure 3** showing PFS for the entire cohort with median PFS of 3.9 months (range: 0-20.1 months.

**Figure 3:**
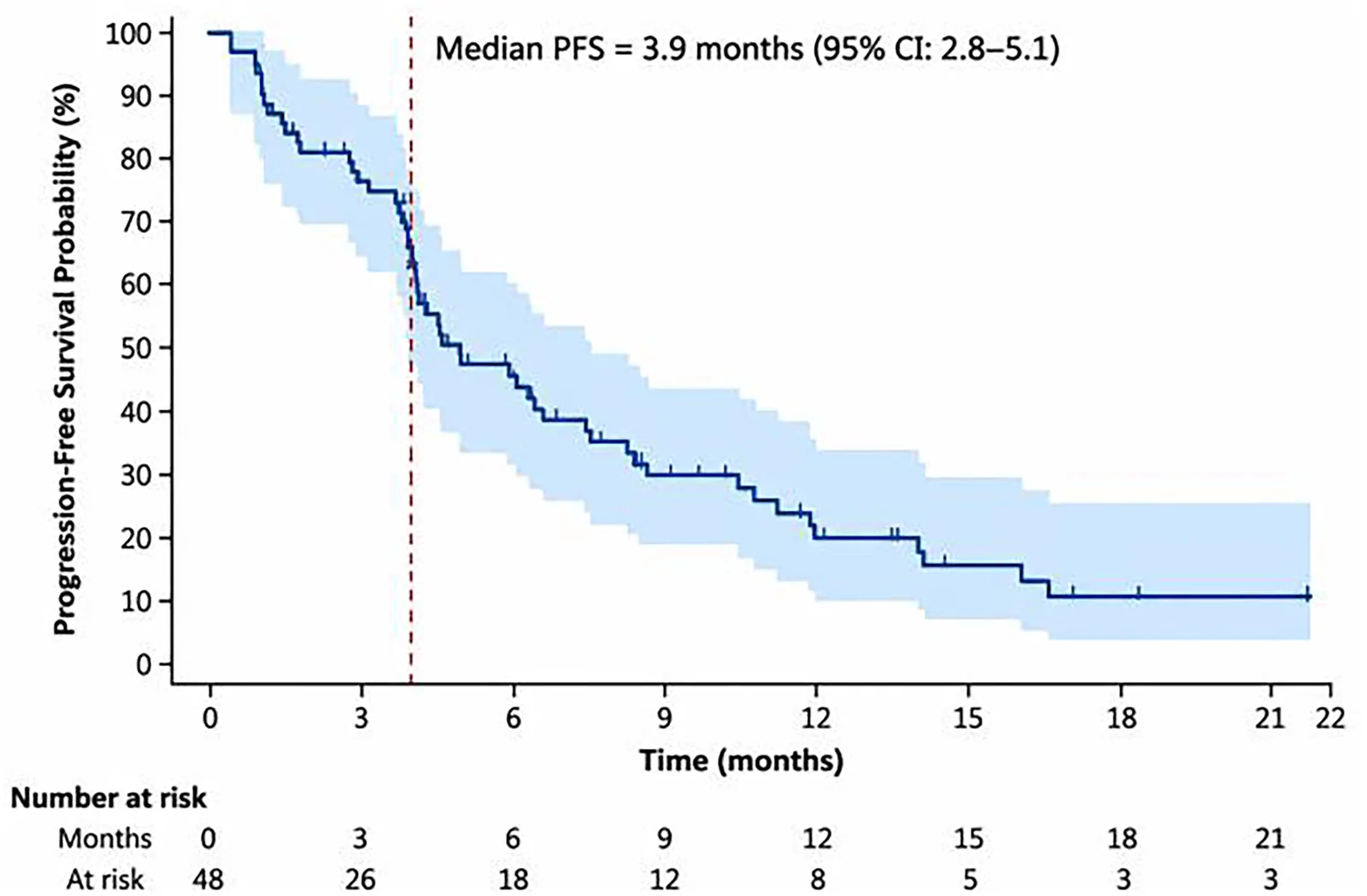
Kaplan-Meier curve - progression-free survival.

The median overall survival was 9.85 months (95% CI: 7.2-12.5), with a range of 2.2-30.7 months. At the time of analysis, 18 patients (37.5%) remained alive, with 8 patients (16.7%) continuing to receive sacituzumab govitecan therapy. The 6-month and 12-month overall survival rates were 78.3% and 45.8%, respectively. **Figure 4** showing survival curve for OS with median OS of 9.85 months.

**Figure 4:**
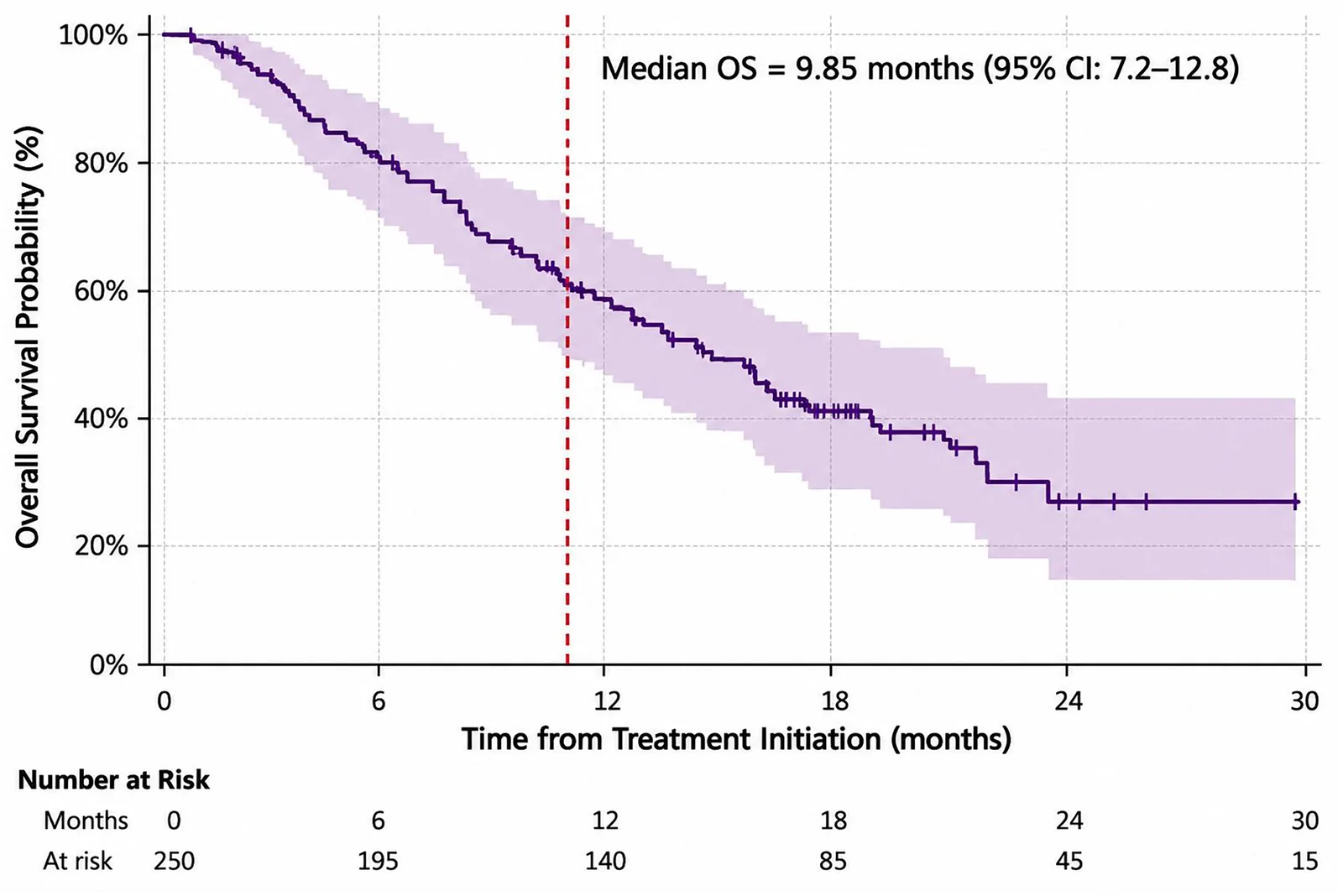
Kaplan-Meier curve - overall survival.

### 2.3 Subgroup analyses

Subgroup analysis by molecular subtype revealed differential outcomes between TNBC and HR+/HER2− cohorts. Patients with TNBC (n=33) achieved a median PFS of 4.2 months compared to 3.1 months in patients with HR+/HER2− disease (n=15), though this difference did not reach statistical significance (p=0.23). Similarly, median OS was 10.2 months in the TNBC group versus 8.9 months in the HR+/HER2− group (p=0.34). **Figure 5** showing PFS stratified by molecular subtype.

**Figure 5:**
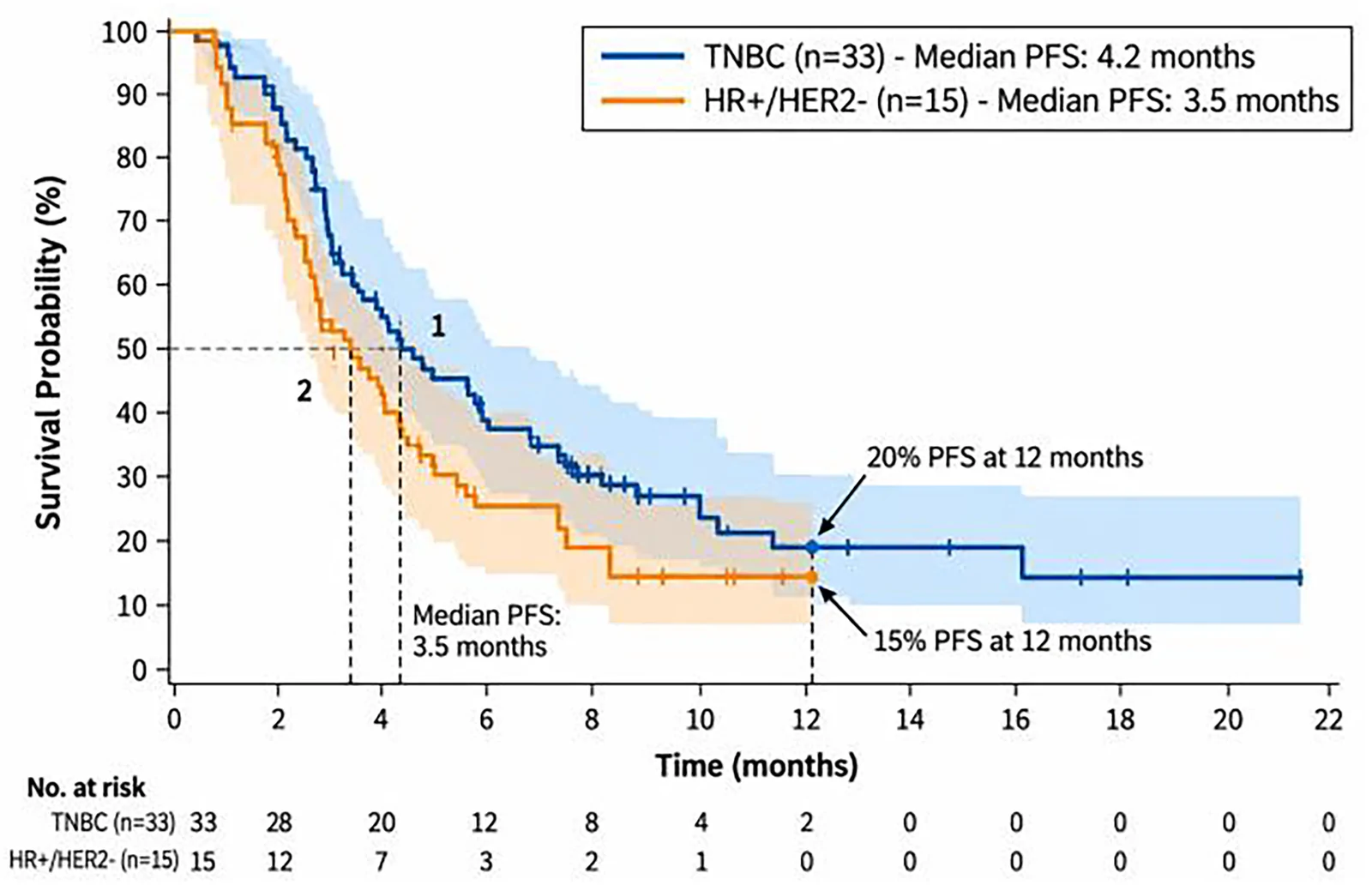
PFS by molecular subtype.

Analysis of systemic outcomes according to CNS metastasis status showed that patients with CNS metastases (n=15) had a median PFS of 3.2 months compared with 4.1 months in patients without CNS involvement (n=33; p=0.18). Median OS was 8.7 months in patients with CNS metastases compared with 10.3 months in those without CNS involvement (p=0.25). These differences were not statistically significant. These analyses reflect systemic PFS and OS and should not be interpreted as evidence of intracranial efficacy. **Figure 6** shows the Kaplan-Meier curves comparing PFS according to CNS metastasis status.

**Figure 6:**
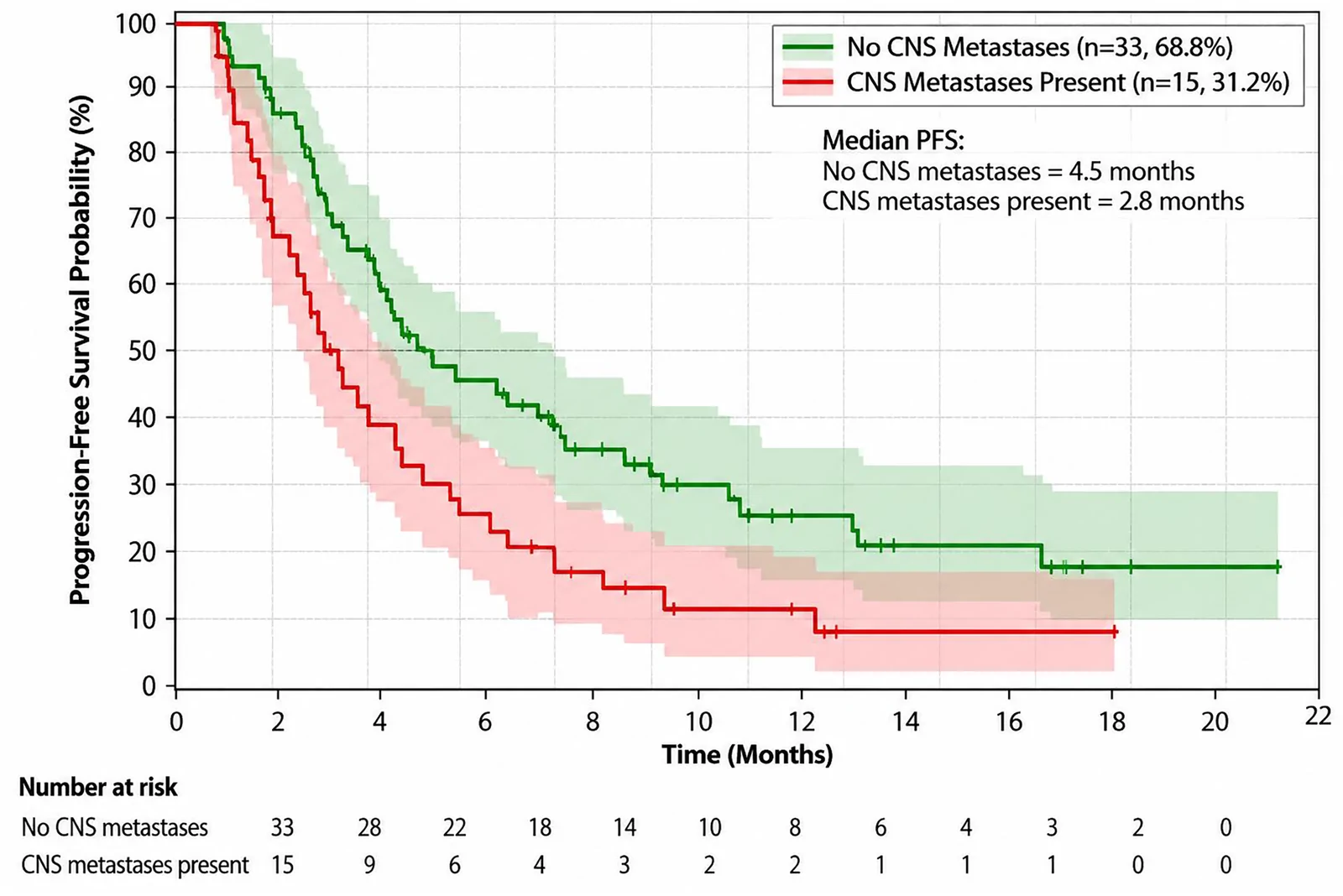
PFS by CNS metastasis status.

### 2.4 Safety and tolerability

Treatment-related adverse events were observed throughout the treatment period, with Grade 3–4 toxicities occurring in 20 patients (41.7%). Grade 3–4 neutropenia occurred in 19 patients (39.6%), followed by rash in 10 patients (20.8%) and pneumonitis in 9 patients (18.7%). Given the relatively high proportion of Grade 3–4 pneumonitis observed in this cohort, this finding warrants cautious interpretation. Because this was a retrospective study, detailed case-level information regarding the radiological characteristics, diagnostic work-up, exclusion of alternative causes, and previous exposure to immune checkpoint inhibitors or other antibody–drug conjugates was not systematically available for all patients with pneumonitis. Therefore, the reported pneumonitis events should be interpreted in the context of the limitations of retrospective adverse-event ascertainment. **Figure 7** shows the frequency of Grade 3–4 adverse events. Hospitalizations related to treatment toxicity occurred in 18 patients (37.5%) and included management of neutropenia, dehydration associated with diarrhea, and clinical monitoring of pneumonitis. However, because detailed case-level clinical information was not systematically captured for each pneumonitis episode, the present study cannot reliably determine the proportion of pneumonitis events directly attributable to sacituzumab govitecan.

**Figure 7:**
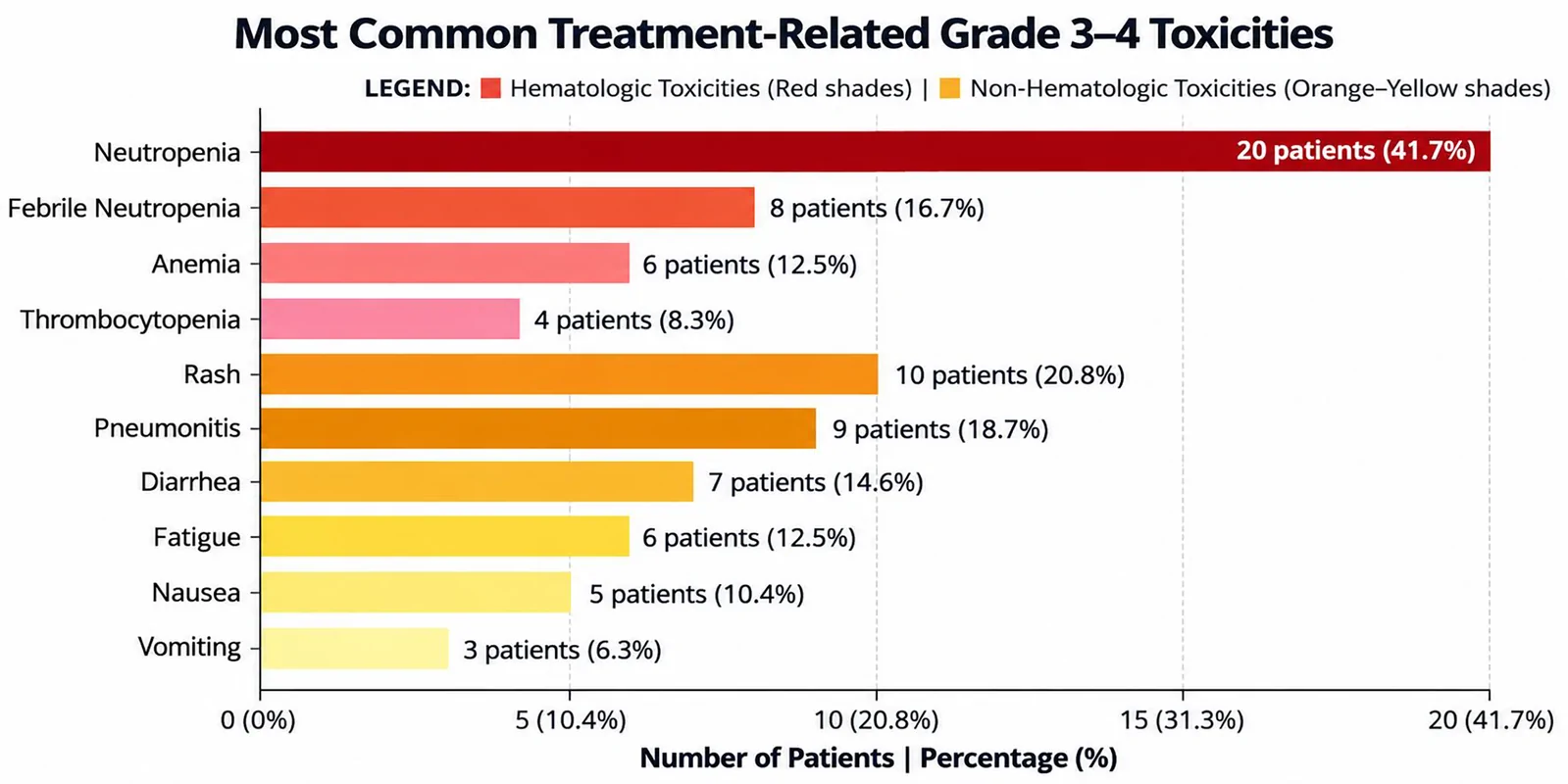
Grade 3–4 adverse events frequency.

Treatment modifications were frequently required to manage toxicities while maintaining treatment delivery. Dose reductions were necessary in 20 patients (41.7%), with the majority able to continue therapy at reduced doses. Treatment delays occurred in 28 patients (58.3%) due to toxicity management or scheduling conflicts (Table A.2). Permanent treatment discontinuation due to adverse events was required in only 6 patients (12.5%), indicating that most toxicities were manageable with appropriate interventions. **Figure 8** illustrating treatment management.

**Figure 8:**
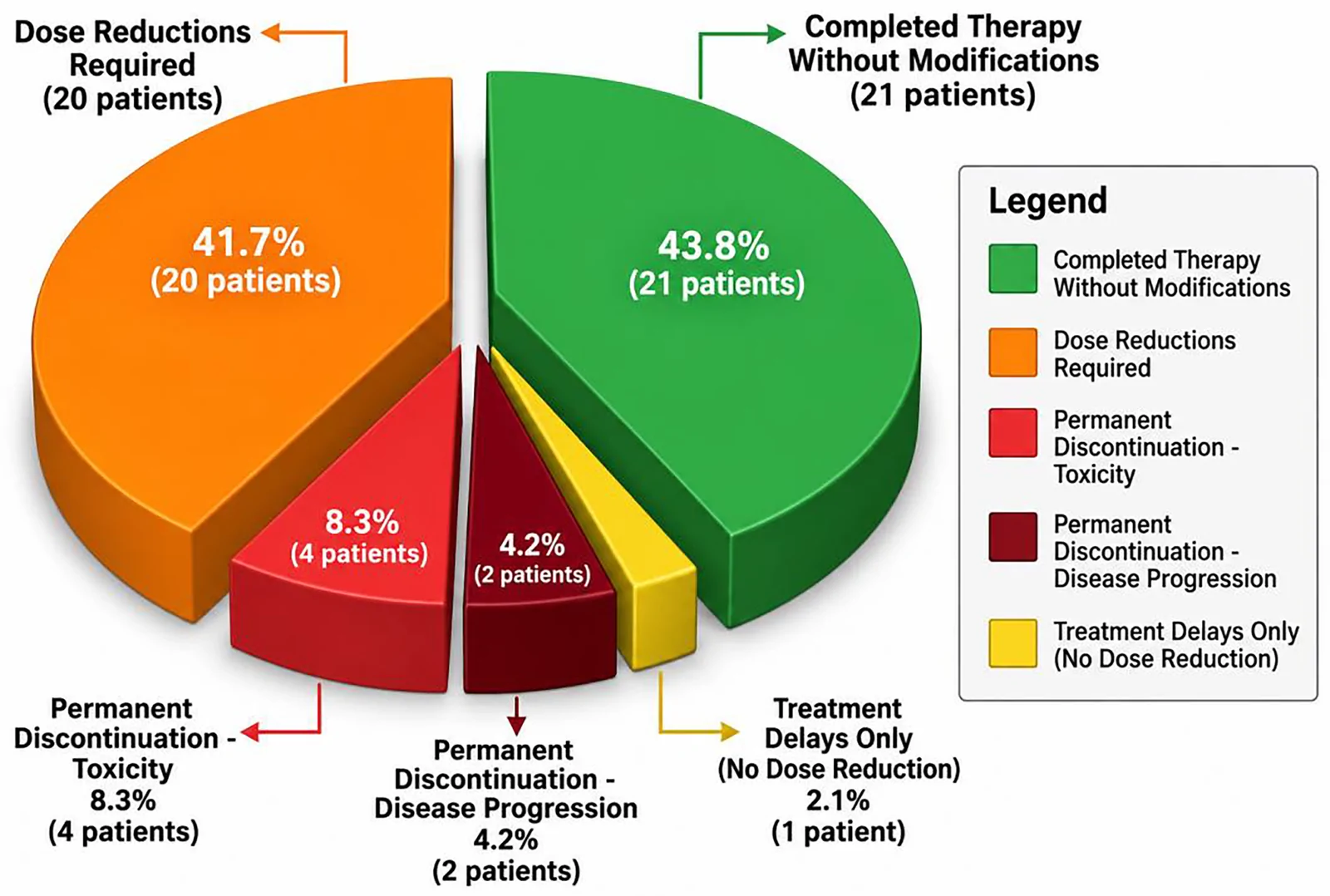
Treatment modifications pie chart.

Hematologic toxicity patterns showed that neutropenia typically occurred early in treatment, with the highest incidence observed during cycles 1-3. Most patients developing severe neutropenia received granulocyte colony-stimulating factor (G-CSF) support, allowing treatment continuation with appropriate monitoring. Febrile neutropenia was uncommon, occurring in only 3 patients (6.3%), and all episodes were successfully managed with hospitalization and supportive care. **Figure 9** showing the incidence of neutropenia over treatment.

**Figure 9:**
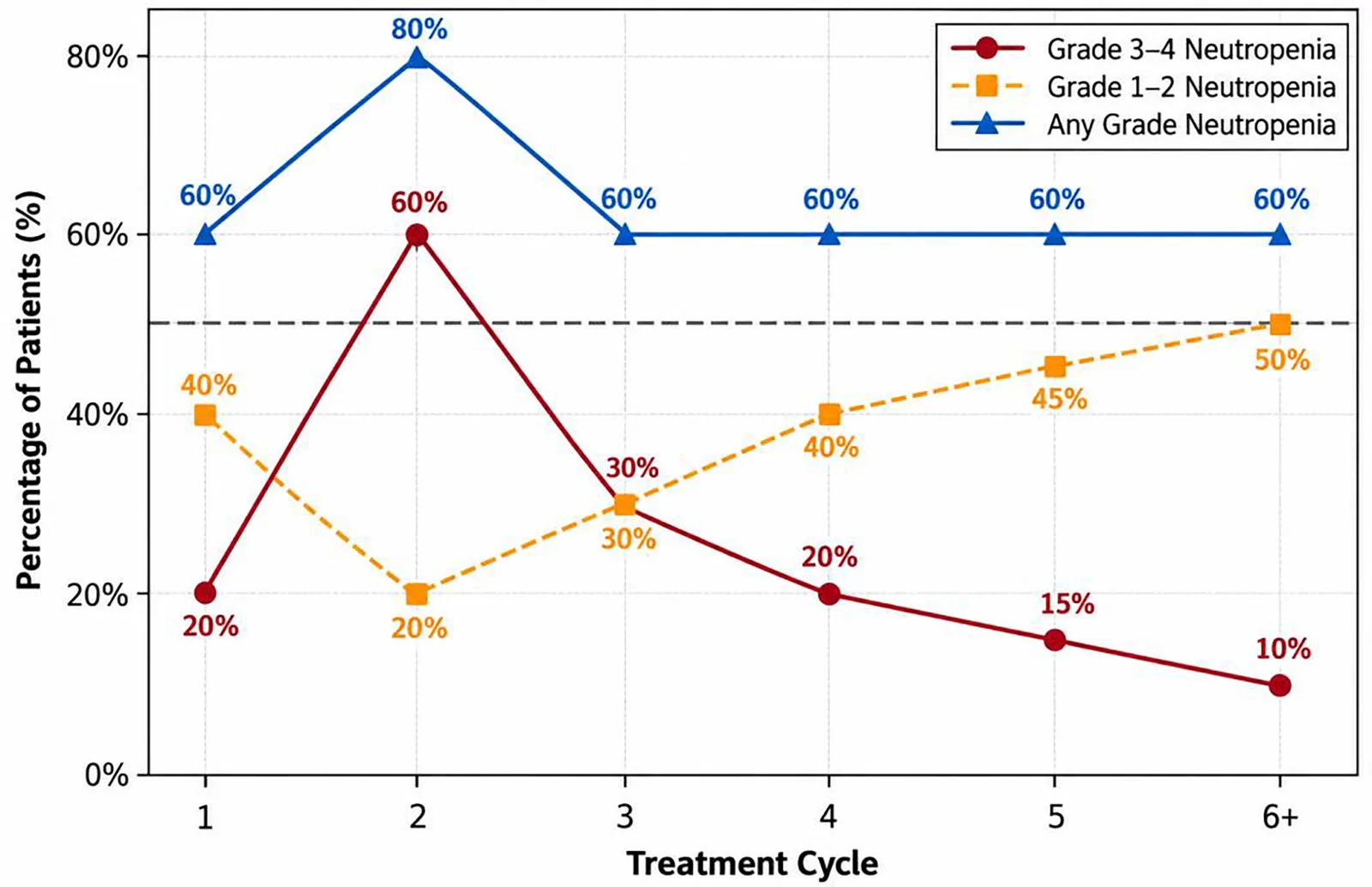
Hematologic toxicity timeline.

Hospitalizations related to treatment toxicity occurred in 18 patients (37.5%), primarily for management of neutropenia, dehydration secondary to diarrhea, or monitoring of pneumonitis. The median duration of hospitalization was 3 days (range: 1–8 days). Supportive care interventions included prophylactic G-CSF in 15 patients (31.3%), topical corticosteroids for rash management in 22 patients (45.8%), and antidiarrheal medications in 35 patients (72.9%). **Figure 10** showing hospitalizations, types of supportive care interventions, topical steroids for rash) and outcomes.

**Figure 10:**
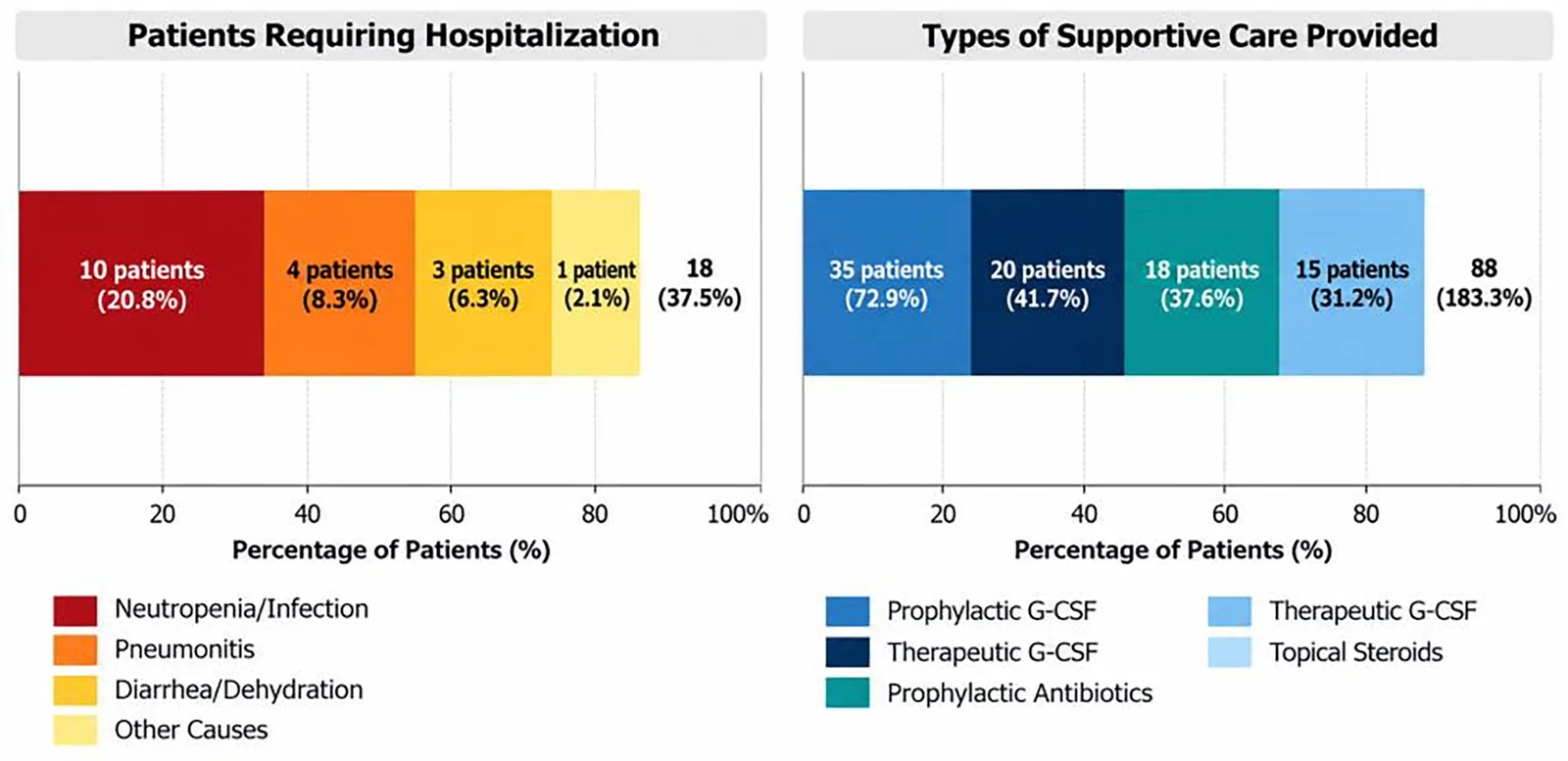
Hospitalizations and supportive care.

### 2.5 Comparative Analysis with Clinical Trials

When compared to pivotal clinical trial data, our real-world cohort demonstrated outcomes that, while numerically lower, remained within clinically meaningful ranges. The median PFS of 3.9 months in our study compared to 5.6 months in the ASCENT trial for TNBC patients, and our median OS of 9.85 months compared to 12.1 months in ASCENT. These differences likely reflect the inclusion of patients with brain metastases, more advanced disease, and potentially lower performance status in our real-world population. **Figure 11** comparing median PFS and OS from this real-world cohort versus ASCENT trial (TNBC) and TROPiCS-02 trial (HR+/HER2-).

**Figure 11:**
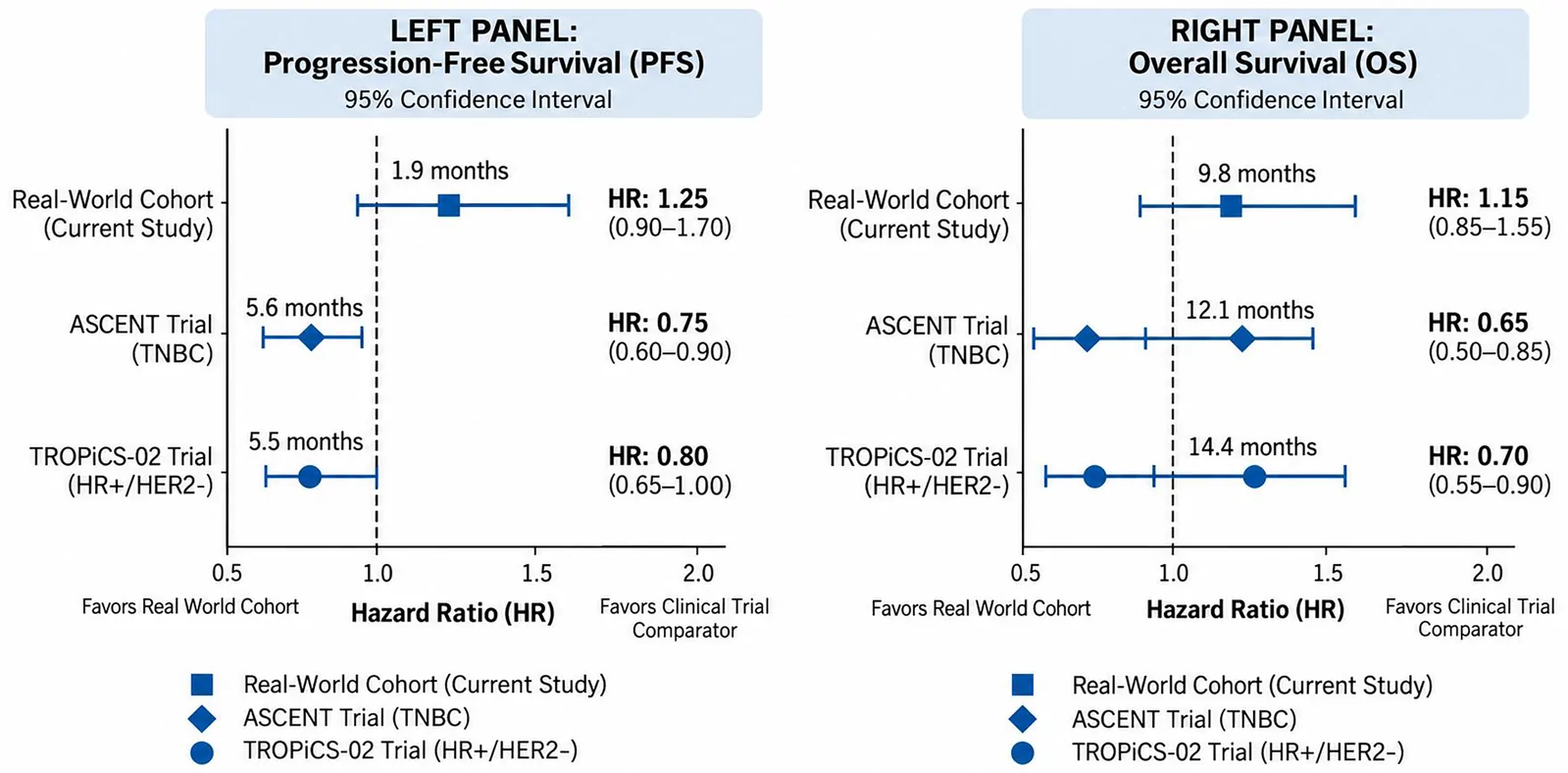
Real-World vs clinical trial outcomes.

Analysis of treatment efficacy by line of therapy revealed maintained benefit across different treatment settings. Patients receiving SG as second-line therapy (n=8) achieved a median PFS of 5.1 months, while those receiving it as third-line (n=18) or fourth-line and beyond (n=22) achieved median PFS of 3.8 and 3.2 months, respectively. This pattern suggests preserved activity even in heavily pretreated populations, though with some attenuation in later treatment lines.

In the multivariable Cox regression analysis, CNS metastasis was associated with a numerically higher hazard of progression or death, although this association was not statistically significant (HR 1.45, 95% CI 0.78–2.71, p=0.24). Patients who had received more than three prior treatment lines had a significantly higher hazard of progression or death compared with patients who had received three or fewer prior treatment lines (HR 1.78, 95% CI 1.02–3.12, p=0.043). Age and molecular subtype were not statistically significantly associated with PFS in the multivariable model. These findings should be interpreted cautiously given the relatively small sample size and retrospective single-center design. **Figure 12** displaying hazard ratios for PFS from multivariable analysis.

**Figure 12:**
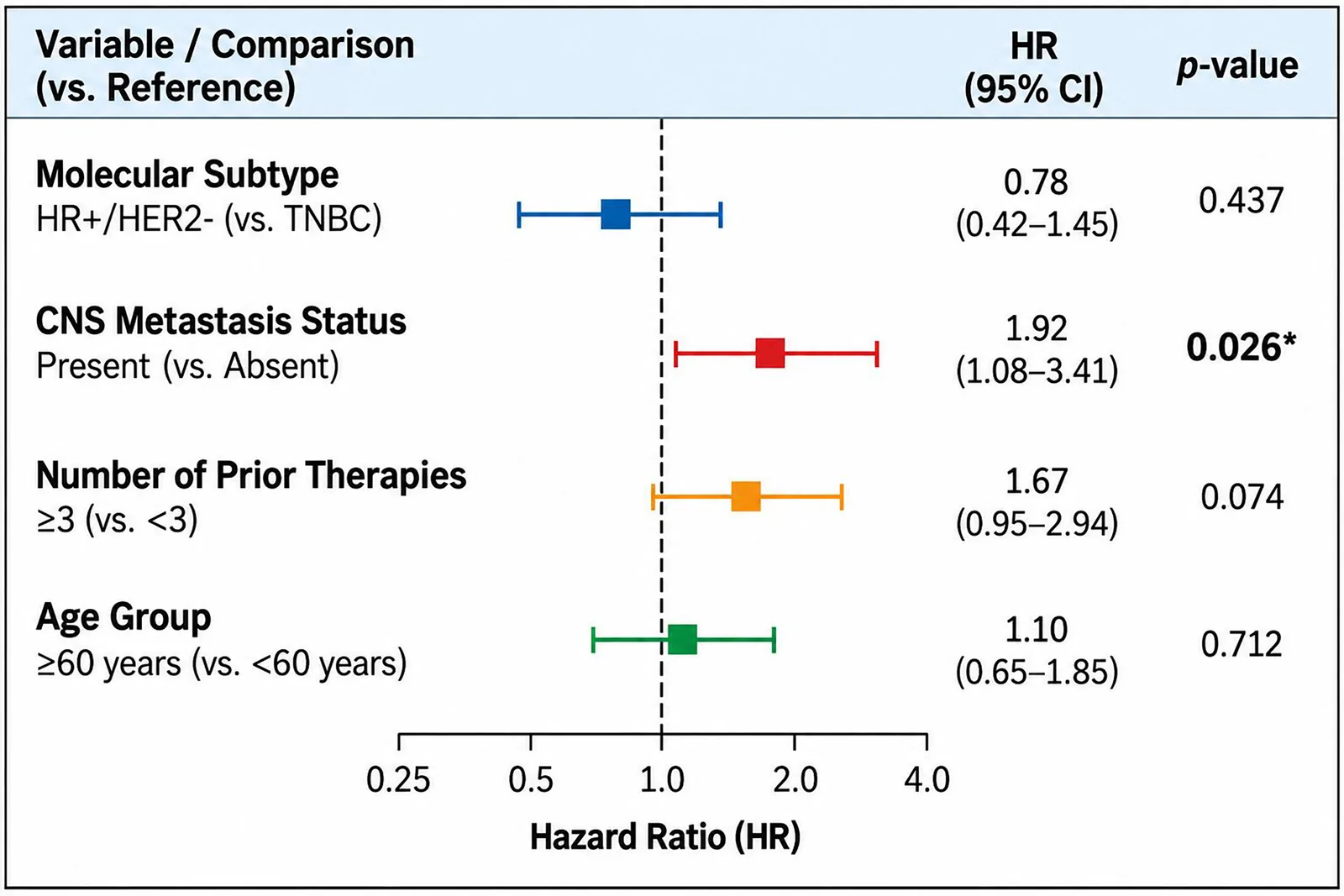
Multivariable Cox proportional hazards regression analysis of progression-free survival.

## 3 Discussion

In this single-center retrospective cohort study, we evaluated the real-world efficacy and safety profile of sacituzumab govitecan in 48 patients with heavily pretreated metastatic breast cancer, including a substantial proportion with brain metastases and diverse molecular subtypes (11, 15). Our findings provide important validation of clinical trial results while extending our understanding of SG’s utility in routine clinical practice, particularly in patient populations often underrepresented in randomized controlled trials (15–17).

The median progression-free survival of 3.9 months and overall survival of 9.85 months observed in our study demonstrate clinically meaningful activity and are reasonably consistent with outcomes reported in prospective clinical trials. In the pivotal ASCENT trial, patients with metastatic TNBC achieved a median PFS of 5.6 months and OS of 12.1 months compared to 1.7 and 6.7 months, respectively, with physician’s choice chemotherapy (11, 14, 17). The somewhat lower survival outcomes in our cohort likely reflect several factors: inclusion of patients with CNS metastases (31.2% versus exclusion in ASCENT), higher rates of heavily pretreated patients, and potentially lower performance status—characteristics commonly encountered in real-world practice but often excluded from registration trials (18–20).

A particularly important aspect of our study is the substantial representation of patients with brain metastases, who comprised nearly one-third of our cohort. This population faces limited therapeutic options and poor prognosis, with most systemic therapies demonstrating limited CNS penetration and efficacy (21). A substantial proportion of our cohort had CNS metastases, with 15 patients (31.2%) having CNS involvement at the initiation of SG. Patients with CNS metastases had a median systemic PFS of 3.2 months and median OS of 8.7 months, compared with 4.1 and 10.3 months, respectively, among patients without CNS involvement. Although these differences were not statistically significant, the present analysis should be interpreted cautiously. Importantly, our study was not specifically designed to evaluate intracranial efficacy, and intracranial response was not assessed as a separate endpoint. Furthermore, the retrospective dataset did not systematically capture the activity or stability of CNS lesions, prior CNS-directed radiotherapy such as stereotactic radiosurgery or whole-brain radiotherapy, or intracranial response independently from systemic disease assessment. Therefore, our findings demonstrate the outcomes of patients with CNS metastases treated with SG in routine practice but do not establish a specific intracranial treatment effect (22).

The safety profile observed in our real-world cohort included both hematologic and non-hematologic toxicities that required active monitoring and treatment modification. Grade 3–4 neutropenia occurred in 19 patients (39.6%), while rash and pneumonitis were reported in 10 (20.8%) and 9 (18.7%) patients, respectively. The relatively high reported frequency of pneumonitis warrants cautious interpretation because the retrospective study design did not allow systematic verification of radiological findings, diagnostic work-up, exclusion of alternative causes, or previous exposure to immune checkpoint inhibitors or other antibody–drug conjugates for every affected patient. Although febrile neutropenia occurred in only 3 patients (6.3%) and supportive care interventions were frequently used, treatment modifications were common (23, 24), with dose reductions required in 20 patients (41.7%), treatment delays occurring in 28 patients (58.3%), and permanent treatment discontinuation due to adverse events in 6 patients (12.5%). These findings emphasize the importance of close toxicity monitoring and individualized supportive care during SG treatment (25).

Our findings also contribute to the evolving understanding of SG activity across varying TROP-2 expression levels and tumor heterogeneity. While TROP-2 expression was not systematically quantified in our retrospective cohort, several patients demonstrated clinical benefit regardless of prior biomarker testing. This observation aligns with emerging preclinical and clinical data suggesting that the potent SN-38 payload may exert significant bystander effects in the tumor microenvironment, potentially extending benefit beyond cells with high TROP-2 expression (12, 26). This mechanism may explain the broad activity observed across different molecular subtypes and treatment histories.

The subgroup analysis by molecular subtype was exploratory, given the imbalance in sample size between the TNBC (n=33) and HR+/HER2− (n=15) cohorts and the differences in treatment context and prior therapy requirements between these populations. Although median PFS and OS were numerically similar between the two subgroups, these findings should not be interpreted as evidence of equivalent efficacy or efficacy independent of hormone receptor status (27, 28). These findings can be considered in the context of the TROPiCS-02 trial, which evaluated SG in heavily pretreated HR+/HER2− metastatic breast cancer. These findings are also consistent with the broader evidence summarized in a recent systematic review and meta-analysis of SG in HR+/HER2− metastatic breast cancer, which included 12 studies and 2,431 patients and reported pooled median PFS and OS of 5.35 and 12.66 months, respectively. However, the substantial heterogeneity across the included studies and the relatively small HR+/HER2− subgroup in our cohort warrant cautious interpretation of cross-study comparisons. Differences in study populations and the small size of our HR+/HER2− subgroup further limit direct comparison. Larger prospective and real-world studies are needed to further characterize the efficacy of SG across molecular subtypes.

Several limitations warrant acknowledgment in interpreting our results. The retrospective, single-institution design introduces potential selection and documentation biases that may influence outcome assessments. Response evaluations were not centrally reviewed, and the timing of imaging studies varied according to clinical practice rather than protocol-specified intervals (29, 30). Additionally, TROP-2 expression levels were not systematically assessed, limiting our ability to correlate biomarker expression with clinical outcomes. Performance status data were incomplete in some patients, preventing comprehensive analysis of this important prognostic factor. Additionally, although CNS metastases were identified using available magnetic resonance imaging or computed tomography documentation, the study did not systematically distinguish active from stable CNS disease or collect detailed information regarding prior CNS-directed interventions, including stereotactic radiosurgery or whole-brain radiotherapy. Intracranial response was also not evaluated as a separate endpoint. Consequently, the observed systemic PFS and OS outcomes in patients with CNS metastases should not be interpreted as evidence of intracranial efficacy. Furthermore, the relatively small sample size limited the number of covariates that could be reliably evaluated in the multivariable survival analysis, and residual confounding cannot be excluded. Therefore, the results of the multivariable Cox regression analysis should be considered exploratory and interpreted cautiously.

Despite these limitations, several factors strengthen the validity and generalizability of our findings. The consecutive patient inclusion reduces selection bias, and the comprehensive data collection from a single institution ensures consistency in treatment delivery, supportive care practices, and follow-up assessments (18, 31). The inclusion of patients with CNS metastases and extensive prior treatment provides information on systemic outcomes with SG in a population often underrepresented in clinical trials and commonly encountered in routine oncology practice. However, because intracranial response was not evaluated as a separate endpoint, these findings should not be interpreted as evidence of specific intracranial efficacy.

Our real-world experience also highlights important practical considerations for SG implementation in clinical practice. The high rate of treatment modifications (41.7% requiring dose reductions) emphasizes the importance of proactive toxicity monitoring and management strategies. Early identification and management of neutropenia, skin toxicity, and gastrointestinal adverse events can optimize treatment delivery while maintaining patient safety and quality of life (32, 33).

### 3.1 Comparison with key trials

Our real-world findings provide valuable validation and context when compared to the pivotal clinical trials that established sacituzumab govitecan as a standard treatment option in metastatic breast cancer. The phase 3 ASCENT trial, which evaluated SG in patients with heavily pretreated metastatic TNBC, demonstrated a median PFS of 5.6 months and median OS of 12.1 months in the SG arm compared to 1.7 and 6.7 months, respectively, with physician’s choice chemotherapy. The confirmed objective response rate was 35%, with a disease control rate exceeding 50%.

In comparison, our real-world cohort achieved a median PFS of 3.9 months and median OS of 9.85 months—outcomes that, while numerically lower, remain within clinically meaningful ranges and reflect the inherent differences between controlled trial populations and real-world practice. The inclusion of patients with brain metastases, more extensive prior treatment exposure, and potentially different performance status distributions in our cohort likely contribute to these differences while demonstrating the broader applicability of SG benefits.

Similarly, the phase 3 TROPiCS-02 trial extended SG’s therapeutic application to HR+/HER2− metastatic breast cancer, showing a median PFS of 5.5 months versus 4.0 months with chemotherapy (HR 0.66; 95% CI: 0.53-0.83; p<0.001), and a median OS of 14.4 months versus 11.2 months (HR 0.79; 95% CI: 0.65-0.96; p=0.020). Although our real-world HR+/HER2− subgroup was relatively small (n=15), the observed trends in disease control and tolerability were consistent with these registration trial findings. Our findings can also be compared with a recent multicenter real-world study conducted by the Turkish Oncology Group, which evaluated 285 patients with unresectable locally advanced or metastatic TNBC treated with SG across 52 oncology centers in Turkey. In that study, median PFS and OS were 5.4 and 12.2 months, respectively, while Grade 3–4 adverse events occurred in 44.2% of patients and dose reductions were required in 20%. Compared with this larger multicenter cohort, our overall median PFS of 3.9 months and median OS of 9.85 months were numerically shorter, while the frequency of Grade 3–4 adverse events in our cohort was comparable. Differences in cohort composition, including the inclusion of HR+/HER2− patients and a substantial proportion of patients with CNS metastases in our study, as well as differences in treatment history and the single-center retrospective design, may contribute to the observed differences. These comparisons should therefore be interpreted descriptively rather than as direct evidence of differences in treatment effectiveness (34).

The safety profiles between our real-world experience and clinical trials showed remarkable consistency. In ASCENT, Grade ≥3 adverse events occurred in 74% of patients receiving SG, with neutropenia (51%), diarrhea (10%), and anemia (8%) being the most common severe toxicities. Our observed rates of Grade 3–4 neutropenia (41.7%) and the overall Grade 3–4 adverse event rate (39.6%) were somewhat lower, possibly reflecting differences in patient monitoring practices, supportive care interventions, or reporting methodologies between clinical trial and real-world settings.

These comparisons underscore several important points: first, the efficacy signals observed in controlled clinical trials translate meaningfully to real-world populations, even when those populations include higher-risk features such as CNS metastases. Second, the safety profile remains manageable in routine practice with appropriate monitoring and supportive care. Third, the broader applicability of SG benefits supports its role as an important therapeutic option across diverse patient populations encountered in clinical practice.

## 4 Materials and methods

This single-center, retrospective cohort study was conducted at Acibadem Maslak Hospital, Department of Medical Oncology, Istanbul, Turkey (35, 36). We identified all consecutive female patients aged ≥18 years with histologically confirmed metastatic breast cancer who received at least one cycle of sacituzumab govitecan between December 2022 and March 2024. Inclusion criteria comprised: (1) pathologically confirmed invasive breast cancer with metastatic disease; (2) treatment with SG as standard of care; (3) adequate baseline organ function; and (4) available follow-up data for efficacy and safety assessment.

Patients were excluded if they had concurrent malignancies, participated in concurrent clinical trials investigating other experimental agents, or had insufficient medical records for analysis. The study was approved by the Institutional Ethics Committee of Acibadem Mehmet Ali Aydinlar University and conducted in accordance with the principles of the Declaration of Helsinki. **Figure 13** showing study flow chart.

**Figure 13:**
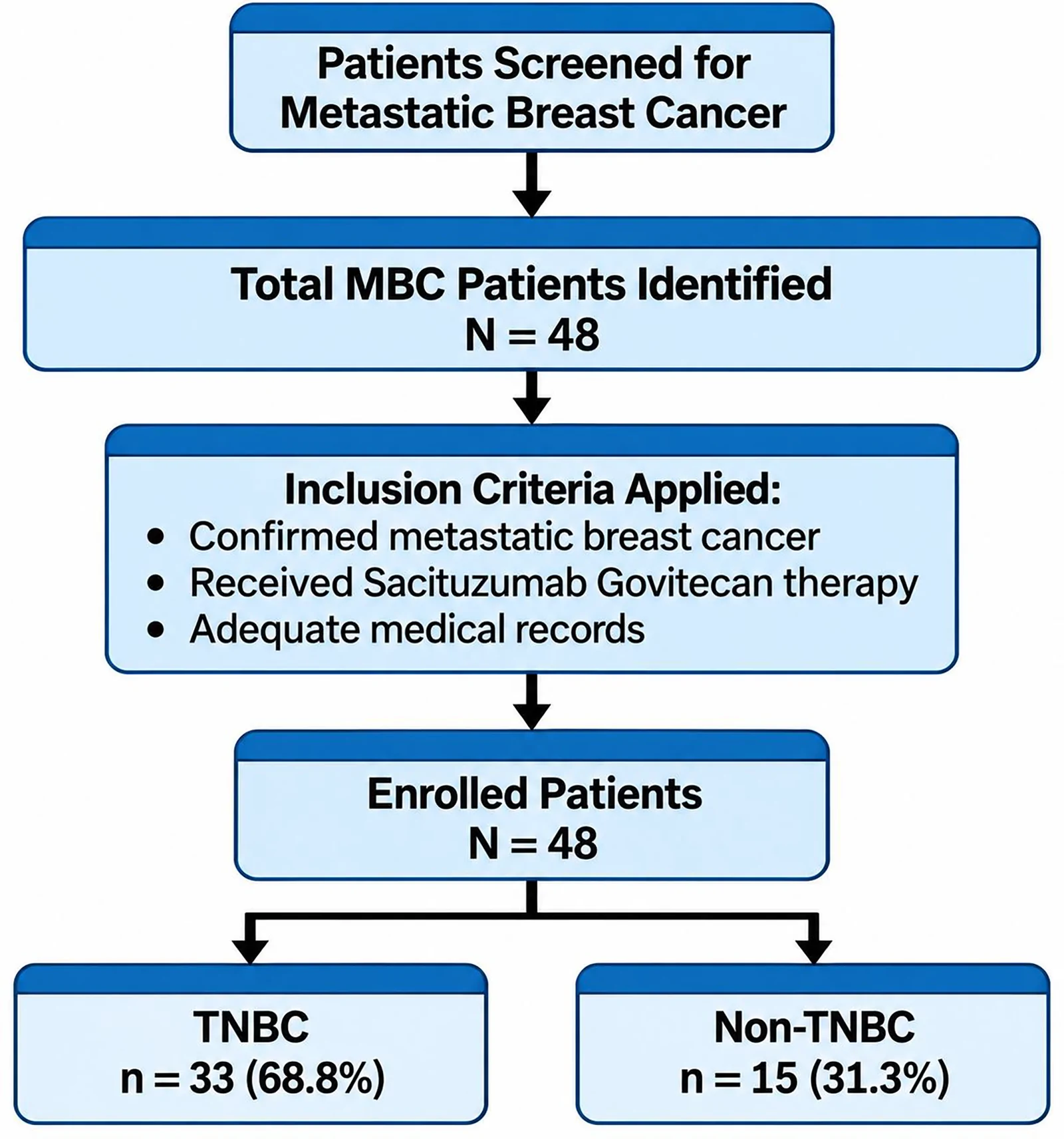
Study flow chart.

Sacituzumab govitecan was administered intravenously at the standard dose of 10 mg/kg on days 1 and 8 of each 21-day cycle, consistent with the approved prescribing information. Treatment continued until disease progression, unacceptable toxicity, patient withdrawal, or physician discretion (11, 37). Dose modifications followed established guidelines, with dose reductions to 7.5 mg/kg and subsequently to 5 mg/kg as clinically indicated. Supportive care measures, including prophylactic antiemetics, growth factor support, and management of treatment-related adverse events, were provided according to institutional standards and clinical judgment (17, 38).

Clinical data were extracted from electronic medical records and included patient demographics, tumor characteristics, molecular subtype classification, staging information, prior treatment history, comorbidities, performance status, and details of SG treatment administration. Molecular subtype classification followed standard definitions: TNBC (estrogen receptor <1%, progesterone receptor <1%, HER2-negative), HR+/HER2− (estrogen or progesterone receptor ≥1%, HER2-negative), and HER2-positive (HER2 3+ by immunohistochemistry or amplified by fluorescence *in situ* hybridization) (11, 39). CNS metastases were identified from available magnetic resonance imaging or computed tomography documentation in the electronic medical records; however, the retrospective dataset did not systematically distinguish active from stable CNS disease or capture the timing and type of prior CNS-directed treatment.

Safety data included all adverse events occurring during treatment and within 30 days of the last SG dose, graded according to the Common Terminology Criteria for Adverse Events (CTCAE) version 5.0. Efficacy endpoints included progression-free survival (PFS), overall survival (OS), objective response rate (ORR), and disease control rate (DCR) (40, 41). Response assessment was performed using Response Evaluation Criteria in Solid Tumors (RECIST) version 1.1 based on routine imaging studies.

Descriptive statistics were used to summarize patient characteristics and treatment outcomes. Categorical variables were presented as frequencies and percentages, while continuous variables were expressed as medians with ranges. Survival outcomes were estimated using the Kaplan-Meier method, with 95% confidence intervals calculated using the Greenwood formula. Comparisons between subgroups were performed using the log-rank test for survival outcomes and Fisher’s exact test or chi-square test for categorical variables, as appropriate. A multivariable Cox proportional hazards regression analysis was performed to evaluate the association between baseline clinical characteristics and progression-free survival. The variables included in the multivariable model were age, molecular subtype, CNS metastasis status, and number of prior treatment lines. Hazard ratios (HRs) with corresponding 95% confidence intervals (CIs) were calculated. The proportional hazards assumption was assessed for the variables included in the Cox regression model. Statistical significance was evaluated using a two-sided p value threshold of less than 0.05.

PFS was defined as the time from SG initiation to disease progression or death from any cause, whichever occurred first. OS was calculated from SG initiation to death from any cause. Patients without events were censored at the last known follow-up date. All statistical analyses were performed using SPSS version 28.0 (IBM Corp., Armonk, NY), with statistical significance defined as p < 0.05 (42, 43).

## 5 Conclusions

*Sacituzumab govitecan* demonstrated clinical activity in this real-world cohort of heavily pretreated patients with metastatic breast cancer. Outcomes were observed across TNBC and HR+/HER2− subgroups and among patients with CNS metastases; however, these subgroup findings were exploratory, and intracranial efficacy was not separately evaluated. The observed toxicity profile, including the need for treatment modifications in a substantial proportion of patients, emphasizes the importance of close monitoring, dose modification, and supportive care during treatment. Larger prospective and multicenter studies are needed to further characterize the efficacy and safety of SG in diverse real-world populations, particularly in patients with CNS metastases.

## Data Availability

The raw data supporting the conclusions of this article will be made available by the authors, without undue reservation.

## Appendix A

### Appendix A.1

**Table A1:** Baseline demographic and clinical characteristics of the study cohort.

Parameter	Value (N = 48)
Median age, years (range)	50.5 (31–69)
Age groups, n (%)	
<40 years	8 (16.7%)
40–49 years	15 (31.2%)
50–59 years	17 (35.4%)
≥60 years	8 (16.7%)
Molecular subtype, n (%)	
Triple-negative	33 (68.8%)
HR+/HER2−	15 (31.2%)
Postmenopausal status, n (%)	33 (68.8%)
*De novo* metastatic disease, n (%)	31 (64.6%)
CNS metastases present, n (%)	15 (31.2%)
Prior treatment exposure, n (%)	
PARP inhibitors	13 (27.1%)
Immunotherapy	24 (50.0%)
CDK4/6 inhibitors	12 (25.0%)
Median prior treatment lines (range)	3 (1-6)

### Appendix A.2

**Table A2:** 2. Grade 3–4 adverse events and treatment modifications.

Adverse event/management parameter	Frequency, n (%)
Grade 3–4 Adverse Events	
Any Grade 3–4 adverse event	20 (41.7%)
Neutropenia	19(39.6%)
Febrile neutropenia	3 (6.3%)
Rash	10 (20.8%)
Pneumonitis	9 (18.7%)
Diarrhea	7 (14.6%)
Fatigue	5 (10.4%)
Treatment Modifications	
Dose reductions required	20 (41.7%)
Treatment delays ≥7 days	28 (58.3%)
Permanent discontinuation due to Aes	6 (12.5%)
Hospitalizations due to Aes	18 (37.5%)
Supportive Care Interventions	
G-CSF support	15 (31.3%)
Topical corticosteroids	22 (45.8%)
Antidiarrheal medications	35 (72.9%)
